# Melatonin attenuates hypoxia-induced epithelial-mesenchymal transition and cell aggressive via Smad7/ CCL20 in glioma

**DOI:** 10.18632/oncotarget.20525

**Published:** 2017-08-24

**Authors:** Xueran Chen, Zhen Wang, Huihui Ma, Shangrong Zhang, Haoran Yang, Hongzhi Wang, Zhiyou Fang

**Affiliations:** ^1^ Cancer Hospital, Chinese Academy of Sciences, Hefei, Anhui, 230031, China; ^2^ Anhui Province Key Laboratory of Medical Physics and Technology, Center of Medical Physics and Technology, Hefei Institutes of Physical Science, Chinese Academy of Sciences, Hefei, Anhui, 230031, China; ^3^ Key Laboratory of Ion Beam Bioengineering, Hefei Institutes of Physical Science, Chinese Academy of Sciences, Hefei, Anhui, 230031, China; ^4^ Department of Radiation Oncology, First Affiliated Hospital, Anhui Medical University, Hefei, Anhui, 230022, China

**Keywords:** melatonin, CCL20, epithelial–mesenchymal transition (EMT), glioma

## Abstract

Tumor recurrence in gliomas is partly attributed to increased epithelial–mesenchymal transition (EMT) and enhanced tumor cell dissemination in the adjacent brain parenchyma. Thus, exploring effective strategies for against EMT-like changes in glioma invasion and recurrence will be important for glioma treatment. In this study, we investigated the roles of melatonin in hypoxia-induced EMT suppression, and found that melatonin could significantly suppress the release of the cytokine, CCL20, from cancer cells and antagonize glioma cell metastasis and invasion under hypoxic stress in glioma cells. Furthermore, our findings show that melatonin deregulates Smad7 expression to suppress TGFβ/Smad-mediated increase in CCL20 transcript levels and CCL20-induced EMT occurrence, suggesting a potential anti-EMT therapeutic role for melatonin in malignant transformation in gliomas.

## INTRODUCTION

Glioma is the most common and lethal type of primary brain tumor [1, 2]. Tumor cells infiltrate into and invade the surrounding normal brain tissues, largely accounting for malignant progression and recurrence of gliomas, especially in glioblastoma multiforme [3, 4].

The tumor microenvironment plays an important role in cancer development and metastasis [5, 6]. Because of rapid tumor cell growth, the tumor often outpaces its blood supply, leading to substantial hypoxia in the vicinity of the tumor [7, 8]. Hypoxia triggers overexpression of hypoxia-inducible factor 1α (HIF-1α), which has previously been shown to induce epithelial–mesenchymal transition (EMT) of cancer cells in various tumors [9, 10]. EMT increases the stem-like characteristics of cancer cells, and converts epithelial cells into motile, invasive mesenchymal cells [11, 12]. Tumor recurrence in glioma is, in part, attributed to increased EMT and enhanced aggressive behavior and treatment resistance of the tumor cells [13, 14]. Therefore, investigation of the molecular mechanisms driving tumor EMT is essential for the development of a curative therapy for glioma patients.

Melatonin (N-acetyl-5-methoxytryptamine), a hormone that is naturally produced and secreted by the pineal gland, has been proven to be effective in tumor inhibition, in both in vitro and in vivo studies [15, 16]. Melatonin inhibits cell viability and proliferation, and induces apoptosis, in breast cancer cells and glioma cells [17, 18]. Studies also suggest that melatonin can induce the degradation of β-catenin, an E-Cadherin repressor, via activation of the kinase protein GSK3β in breast cancer cells [19]. Recent studies have reported that melatonin can suppress hypoxia-induced glioblastoma cell migration and invasion [20, 21]. These findings indicate a potential inhibitory role of melatonin on hypoxia-induced EMT in glioma, but the mechanism still remains elusive.

Accumulating evidence showed that few chemokines derived from tumor-associated leukocytes or tumor cells acted as growth factors for cancer cells, and could contribute to tumor metastasis by accelerating angiogenesis, attracting endothelial cells or regulating the motility of cancer cells [22, 23]. Among these, CCL20 has been proven to be related to invasion and metastasis in some types of cancer [24, 25]. CCL20 had been reported to induce EMT, and is correlated with tumor formation, metastasis, or progression in many malignant neoplasms such as colorectal cancer and hepatocellular carcinoma [26, 27]. However, the relationship between CCL20 and melatonin-mediated anti-EMT in glioma cells remains unclear.

In this study, we explore the interaction between melatonin-antagonized EMT and tumor micro-environment. We show that increased levels of CCL20 released from cancer cells significantly induce EMT under hypoxic stress in glioma cells, while melatonin can reverse this transition. Furthermore, our findings show that melatonin deregulates Smad7 expression to suppress TGFβ/Smad-mediated increase in CCL20 transcript levels and CCL20-induced EMT occurrence, suggesting potential anti-EMT therapeutic strategy of melatonin to overcome malignant transformation in gliomas.

## RESULTS

### Melatonin inhibits hypoxia-mediated EMT in glioma

**Figure 1:**
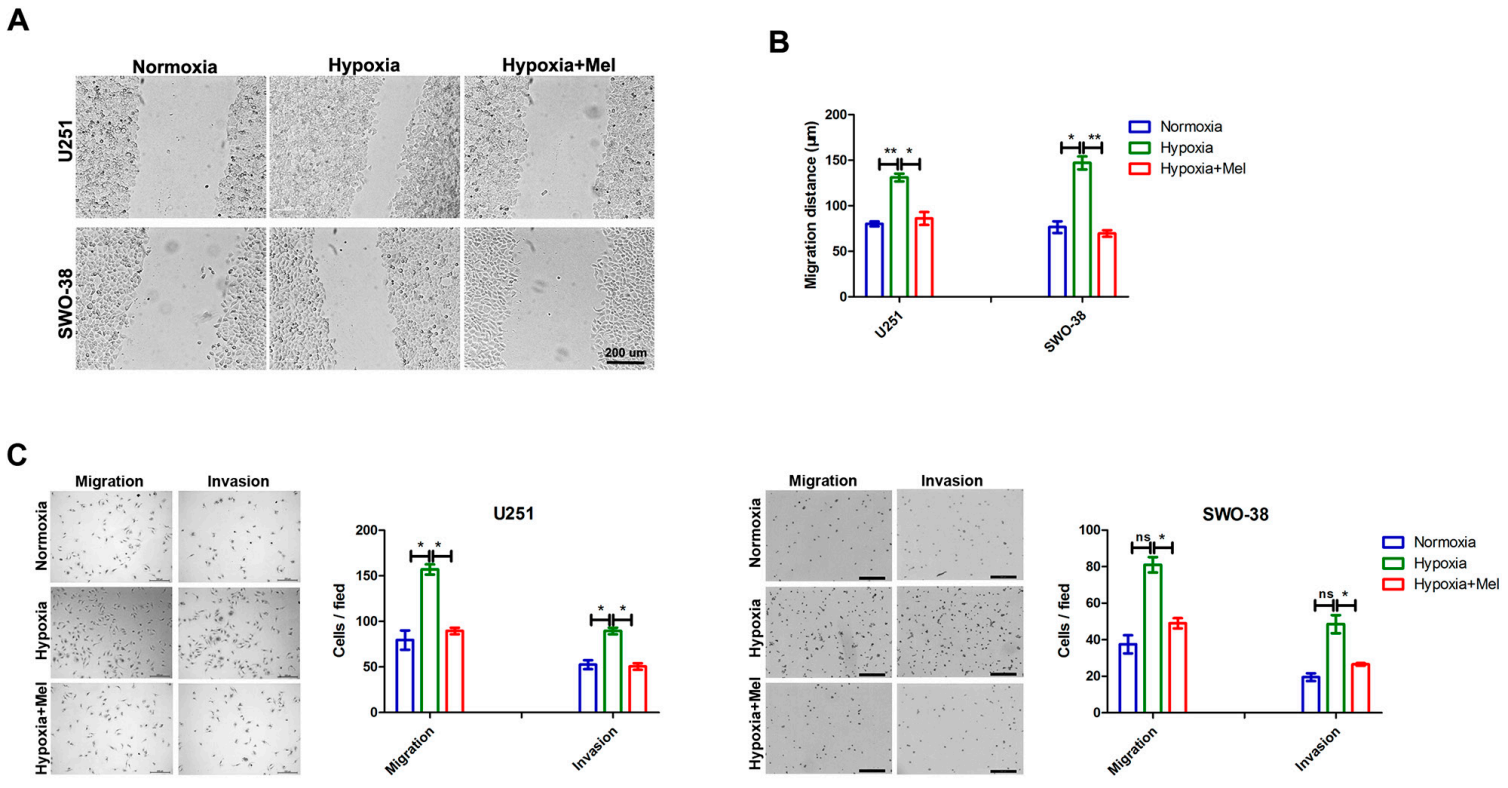
Effect of melatonin on migration and invasion of glioma cells induced by hypoxia **(A)** U251 and SWO-38 cells scraped by a pipette tip were incubated with or without 1mM melatonin and then exposed to hypoxia or normoxia for 18 hours. A representative of wound healing assay was presented. Scale bar=200 um. **(B)** The quantification of U251 and SWO-38 cells migration areas was performed. The shown data represent the mean±SD of triplicate determinations from three separate experiments and compared using the unpaired *t* test (^*^, P<0.05; ^**^, P<0.01). **(C)** The migration and invasion abilities of U251 and SWO-38 cells were determined by Boyden Chamber Transwell assays. The cell migration and invasion abilities were quantified. Mean cell counts from at least 10 fields and data represent the mean±SD of triplicate determinations from three separate experiments and compared using the unpaired *t* test (ns, not significant; ^*^, P<0.05). Mel, melatonin.

As reported earlier, melatonin can suppress hypoxia-induced glioma cell migration and invasion [20, 21, 28]. The migratory activities of both U251 and SWO-38 glioma cells that were exposed to hypoxia were evidently increased as compared to those of the normoxic controls; the increase was significantly blocked by 1 mM melatonin (Figure 1A). Statistical analysis showed that melatonin drastically suppressed cell migration of both U251 (48 % reduction; p<0.05) and SWO-38 (55 %; p<0.01) cell lines as compared with untreated control cells (Figure 1B). To preclude the possibility that the reduced cell migration upon melatonin treatment was associated with decreased cell proliferation, we also assessed the migration and invasion rate of U251 and SWO-38 using Transwell assays under either normoxic or hypoxic conditions for 18 h (Figure 1C). Hypoxic cells (2 × 10^5^/well) exhibited a significantly increased capacity to penetrate the chamber filter, which was reversed by melatonin pretreatment. Thus, these results suggest that melatonin suppresses the cell motility and metastasis of glioma cells under hypoxic stress.

**Figure 2:**
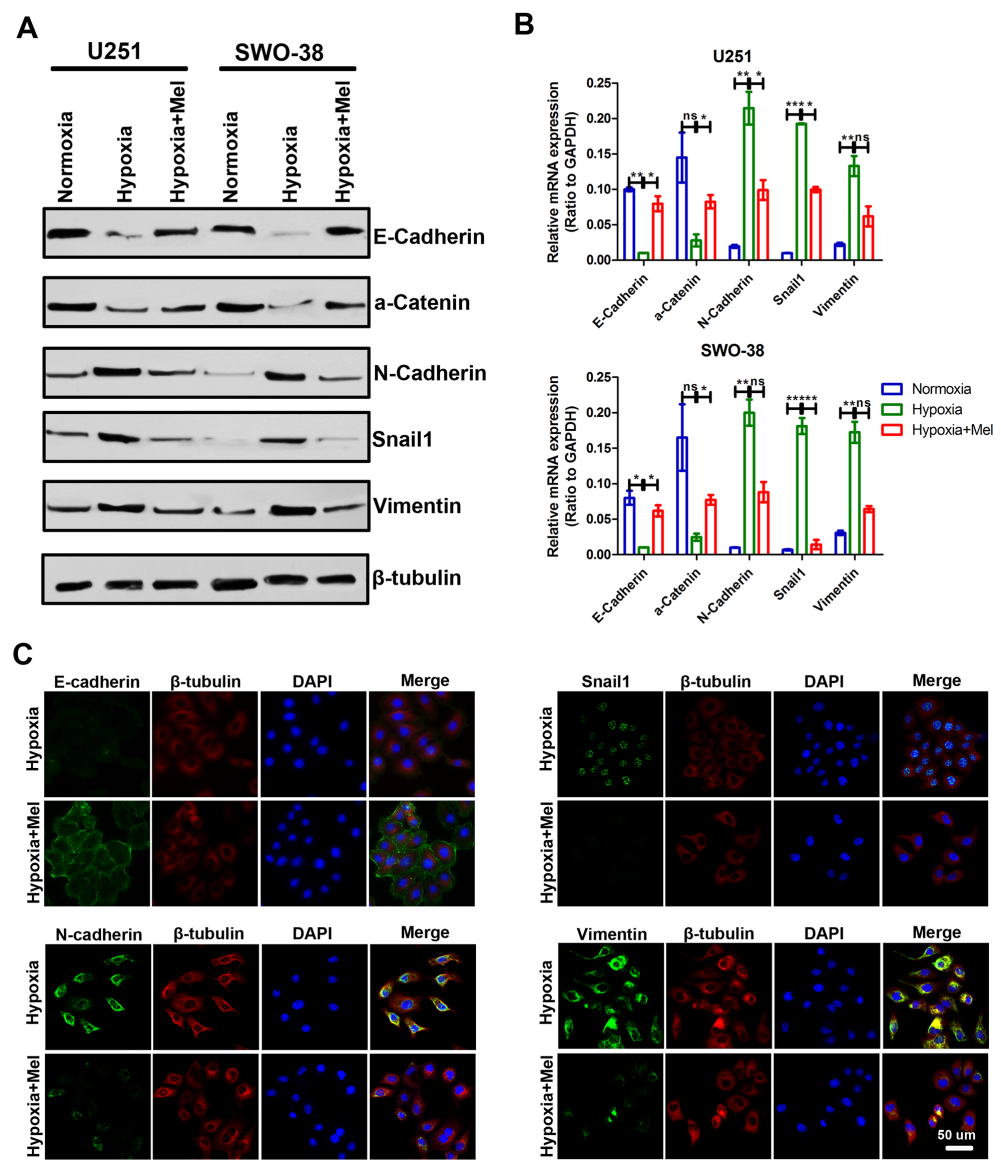
Effect of melatonin on the transition between epithelial and mesenchymal phenotypes in glioma cells under hypoxic stress **(A)** The expression of epithelial (E-Cadherin and a-Catenin) and mesenchymal (N-Cadherin, Snail1, and Vimentin) markers was analyzed by Western blot analysis in U251 and SWO-38 cells treated with or without melatonin under hypoxia. β-tubulin was used as loading control. **(B)** The expression of epithelial (E-Cadherin and a-Catenin) and mesenchymal (N-Cadherin, Snail1, and Vimentin) markers was analyzed by qRT-PCR in U251 and SWO-38 treated with or without melatonin under hypoxia. All values were normalized to GAPDH expression. The shown data represent the mean±SD of triplicate determinations from three separate experiments and compared using the unpaired *t* test (ns, not significant; ^*^, P<0.05; ^**^, P<0.01; ^***^, P<0.001). **(C)** The expression of E-Cadherin, N-Cadherin, Snail1 and Vimentin was analyzed by immunofluorescence in SWO-38 cells treated with or without melatonin under hypoxia. Scale bar=50 um.

The metastatic cascade represents a multi-step process, in which EMT is a crucial event in the early stages of cancer metastasis [29–31]. Accordingly, exposure of U251 and SWO-38 cells to hypoxia for 24 h led to E-Cadherin and α-Catenin downregulation (Figure 2A). Concomitantly, hypoxia induced the expression of the mesenchymal markers, Vimentin, N-Cadherin, and Snail1. However, melatonin drastically inhibited hypoxia-induced expression of Vimentin, N-Cadherin, and Snail1, and reversed the E-Cadherin and α-Catenin levels. Moreover, the shift in expression levels of mRNA was correlated with the corresponding protein levels (Figure 2B). This result suggests that melatonin affects the expression of epithelial and mesenchymal markers at the transcript level. A similar result was also obtained using immunofluorescence analyses (Figure 2C).

### Melatonin suppresses hypoxia-mediated stem cell self-renewal in glioma

**Figure 3:**
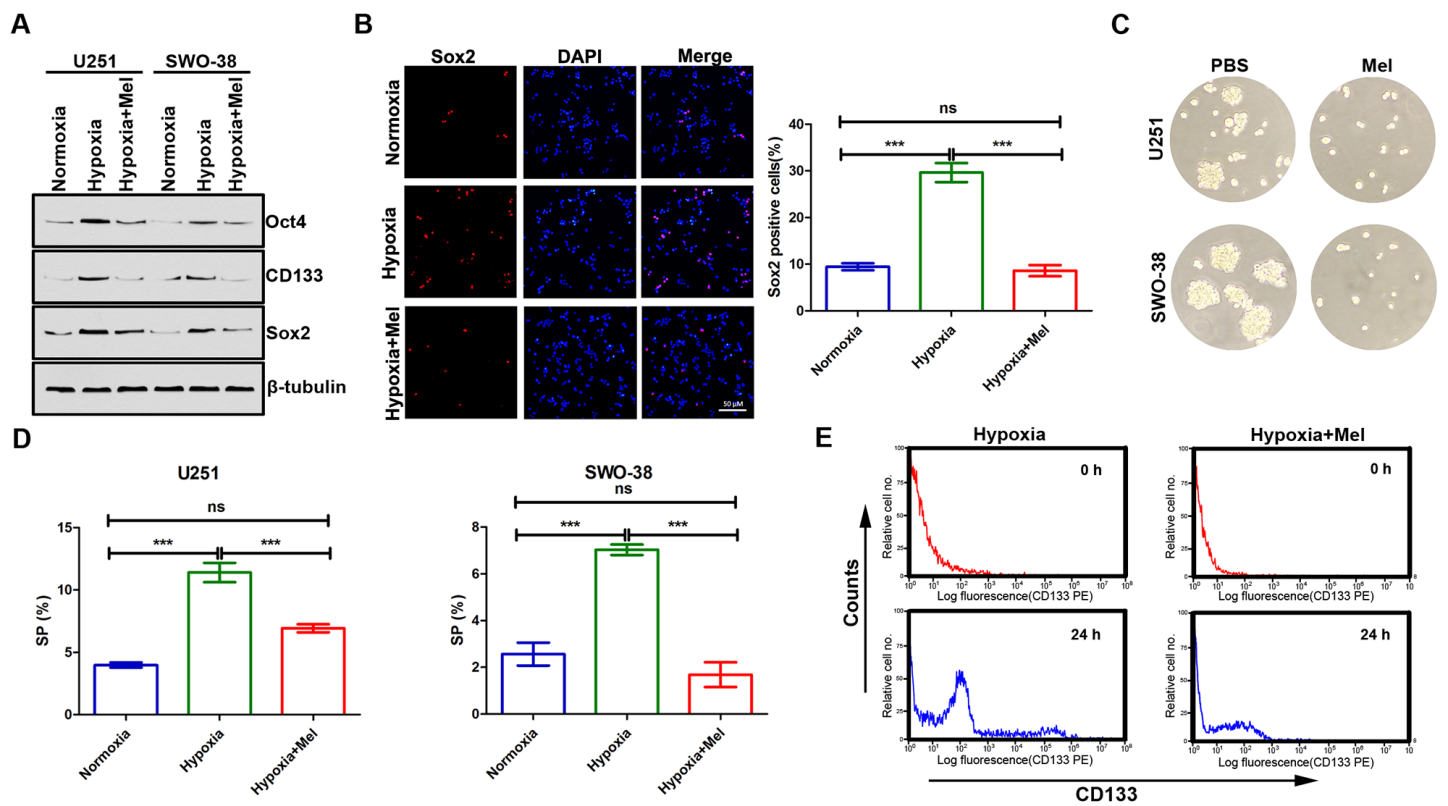
Effect of melatonin on cancer stem cell self-renewal in glioma cells under hypoxic stress **(A)** The expression of stemness markers (Oct4, CD133 and Sox2) was analyzed by Western blot analysis in U251 and SWO-38 cells treated with or without melatonin under hypoxia. β-tubulin was used as loading control. **(B)** The expression of Sox2 was analyzed by immunofluorescence in SWO-38 cells treated with or without melatonin under hypoxia. Scale bar=50 um. The percentage of Sox2 positive cells was quantified. Mean cell counts from at least 5 fields and data represent the mean±SD of triplicate determinations from three separate experiments and compared using the unpaired *t* test (ns, not significant; ^***^, P<0.001). **(C)** The sphere formation was determined by colony assays in U251 and SWO-38 cells treated with or without melatonin under hypoxia. **(D)** The SP population was determined by Hoechst 33342 efflux assays in U251 and SWO-38 cells treated with or without melatonin under hypoxia. The SP population was quantified. The shown data represent the mean±SD of triplicate determinations from three separate experiments and compared using the unpaired *t* test (ns, not significant; ^***^, P<0.001). **(E)** The expression and secretion of CD133 were detected by membrane-labeling experiments in SWO-38 cells treated with or without melatonin under hypoxia.

Increasing evidence has linked EMT with the acquisition of molecular and functional traits of stem cells in normal and neoplastic cell populations [32, 33]. Thus, we attempted to determine whether melatonin regulates certain stem cell associated properties. Hypoxia promoted expression of stemness markers, and holoclone formation in U251 and SWO-38 cells (Figure 3A–3C). In contrast, the presence of melatonin inhibited these capacities. Previous studies have shown that side population (SP) cells have stem cell characteristics and enrich the stem cell population. In this study, we also found that hypoxia stress increased the percentage of SP cells, and melatonin reduced the abundance of SP cells (Figure 3D). The role of melatonin on stem cell self-renewal in U251 was also determined by membrane-labeling experiments in suspension culture (Figure 3E). Cells treated with trypsin were labeled using CD133 fluorescent-antibody. Fluorescence-activated cell sorting (FACS) analysis revealed that hypoxia treated cells recovered fluorescence more rapidly after 24 h of culture than melatonin treated cells did, indicating that melatonin suppresses hypoxia-mediated stem cell self-renewal in glioma.

### Melatonin inhibits glioma tumorigenic potential through EMT blocking

**Figure 4:**
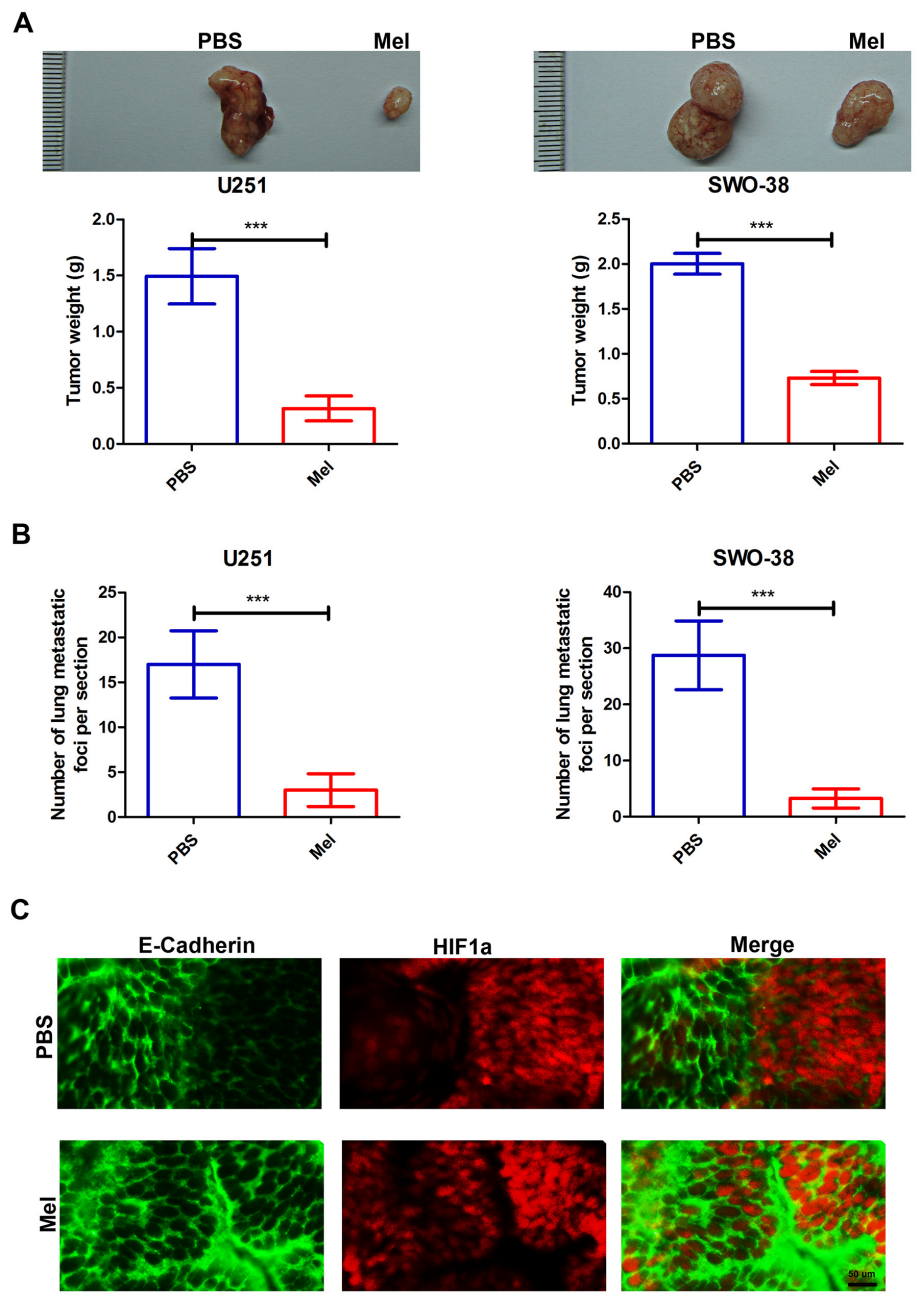
Effect of melatonin on metastasis capacities of glioma cells *in vivo* **(A)** representative images (upper), and weight (lower) of tumors following subcutaneous injection of U251 and SWO-38 cells. Nude mice were injected melatonin at 10mg/kg every 5 days through the tail vein. The weight of tumors was quantified. The shown data represent the mean±SD of triplicate determinations from five separate experiments and compared using the unpaired *t* test (^***^, P<0.001). **(B)** The number of metastatic foci per section in lungs was quantified from individual mouse with injection of U251 and SWO-38 cells. The shown data represent the mean±SD of triplicate determinations from five separate experiments and compared using the unpaired *t* test (^***^, P<0.001). **(C)** The expression of hypoxic marker (HIF1a) and epithelial marker (E-Cadherin) was analyzed by immunofluorescence in tumor slices from mouse with injection of SWO-38 cells. Scale bar=50 um.

To extend our *in vitro* observations, we investigated whether melatonin regulates the tumorigenic and metastasis capacity of glioma cells *in vivo*. U251 and SWO-38 cells were subcutaneously injected into nude mice, and mice were injected with 10 mg/kg of melatonin every 5 days through the tail vein. Melatonin remarkably decreased the tumor volume and weight (Figure 4A), and dramatically reduced the number of tumors in the lung of each mouse (Figure 4B). To further study the relationship between the effect of melatonin on glioma tumorigenic potential and EMT, the mice were sacrificed and the tumors were excised. Slices of the glioma were stained with anti-E-Cadherin and anti-HIF1a antibodies and analyzed by confocal fluorescence microscopy. As shown in Figure 4C, hypoxic tumor regions (indicated by HIF1a) showed reduced E-Cadherin levels, but melatonin treatment visibly increased E-Cadherin localization at the cell membrane. Therefore, the *in vivo* studies further demonstrated the important therapeutic effect of melatonin in controlling glioma tumorigenic potential through EMT blocking.

### CCL20 is a mediator of melatonin-mediated EMT in glioma

**Figure 5:**
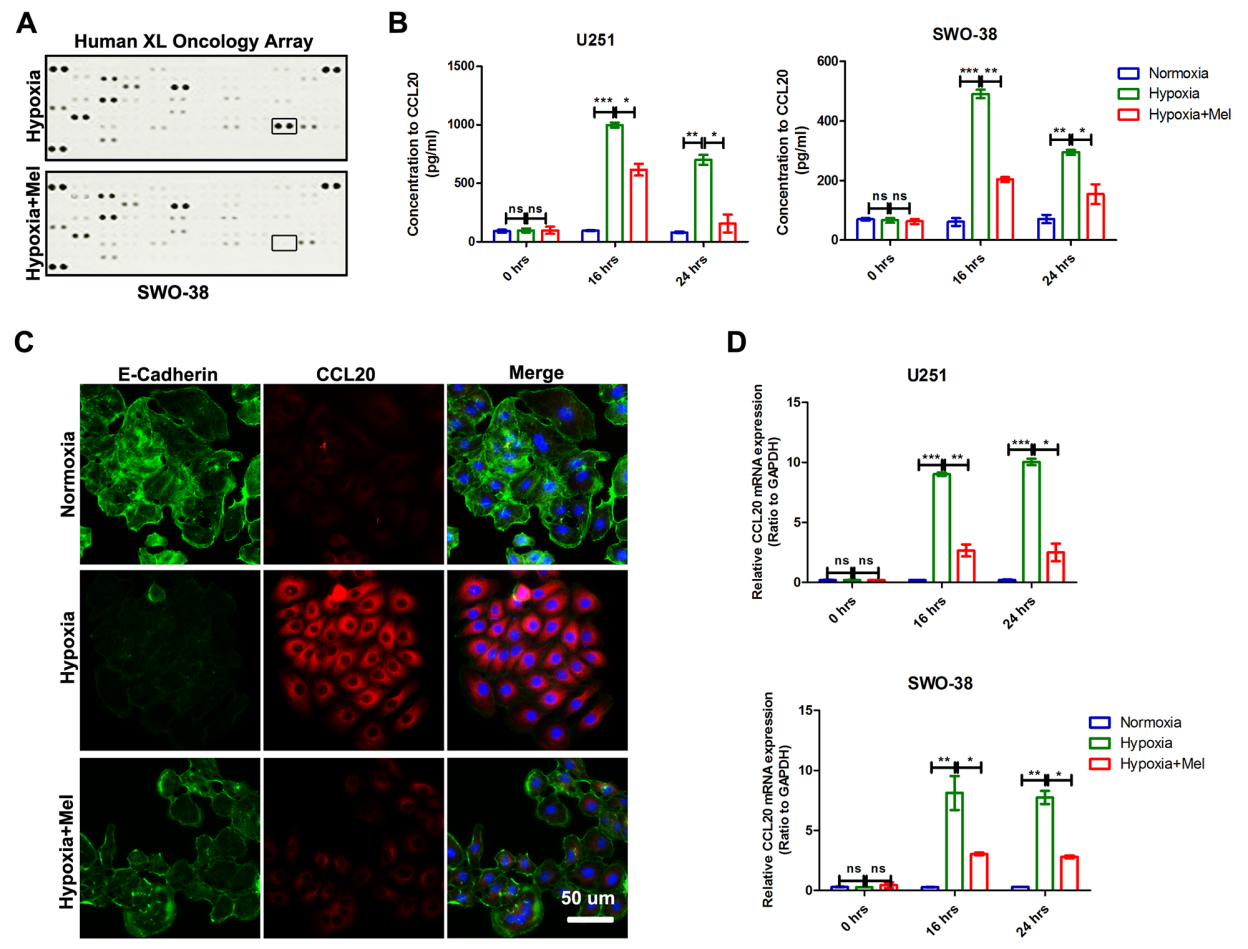
Effect of melatonin on CCL20 expression and secretion level in glioma cells under hypoxic stress **(A)** The change in the cytokine profile associated with melatonin-mediated EMT of glioma cells in hypoxia was detected by The Human XL Oncology Array. CCL20, indicated by a square frame. **(B)** The secretion level of CCL20 was determined by ELSA in U251 and SWO-38 cellstreated with or without melatonin under hypoxia. The shown data represent the mean±SD of triplicate determinations from three separate experiments and compared using the unpaired *t* test(ns, not significant; ^*^, P<0.05; ^**^, P<0.01; ^***^, P<0.001). **(C)** The expression of CCL20 and E-Cadherin was determined by immunofluorescence in SWO-38 cells treated with or without melatonin under hypoxia. Scale bar=50 um. **(D)** The expression of CCL20 was analyzed by qRT-PCR in U251 and SWO-38 treated with or without melatonin under hypoxia from 0 to 24 hours. CCL20 mRNA level was normalized to GAPDH expression. The shown data represent the mean±SD of triplicate determinations from three separate experiments and compared using the unpaired *t* test (ns, not significant; ^*^, P<0.05; ^**^, P<0.01; ^***^, P<0.001).

Cytokine profiles within the tumor micro environment play an important role in cancer development and metastasis [34, 35]. To investigate whether there was a change in the cytokine profile associated with melatonin-mediated EMT of glioma cells in hypoxia, the medium from SWO-38 cells was analyzed using the human XL oncology array kit (ARY026; R&D Systems) (Figure 5A). The levels of CCL20 decreased most dramatically in the presence of melatonin. ELISA studies also confirmed the inhibitory effect of melatonin on CCL20 accumulation in U251 and SWO-38 (Figure 5B). In addition, immunofluorescence staining showed that hypoxia induced CCL20 expression and decreased E-Cadherin level, but melatonin could reverse these changes (Figure 5C). We used qRT-PCR to confirm the increase in *CCL20* expression at 16 and 24 h in both U251 cells and SWO-38 cells under hypoxic conditions, which was blocked by melatonin.

**Figure 6:**
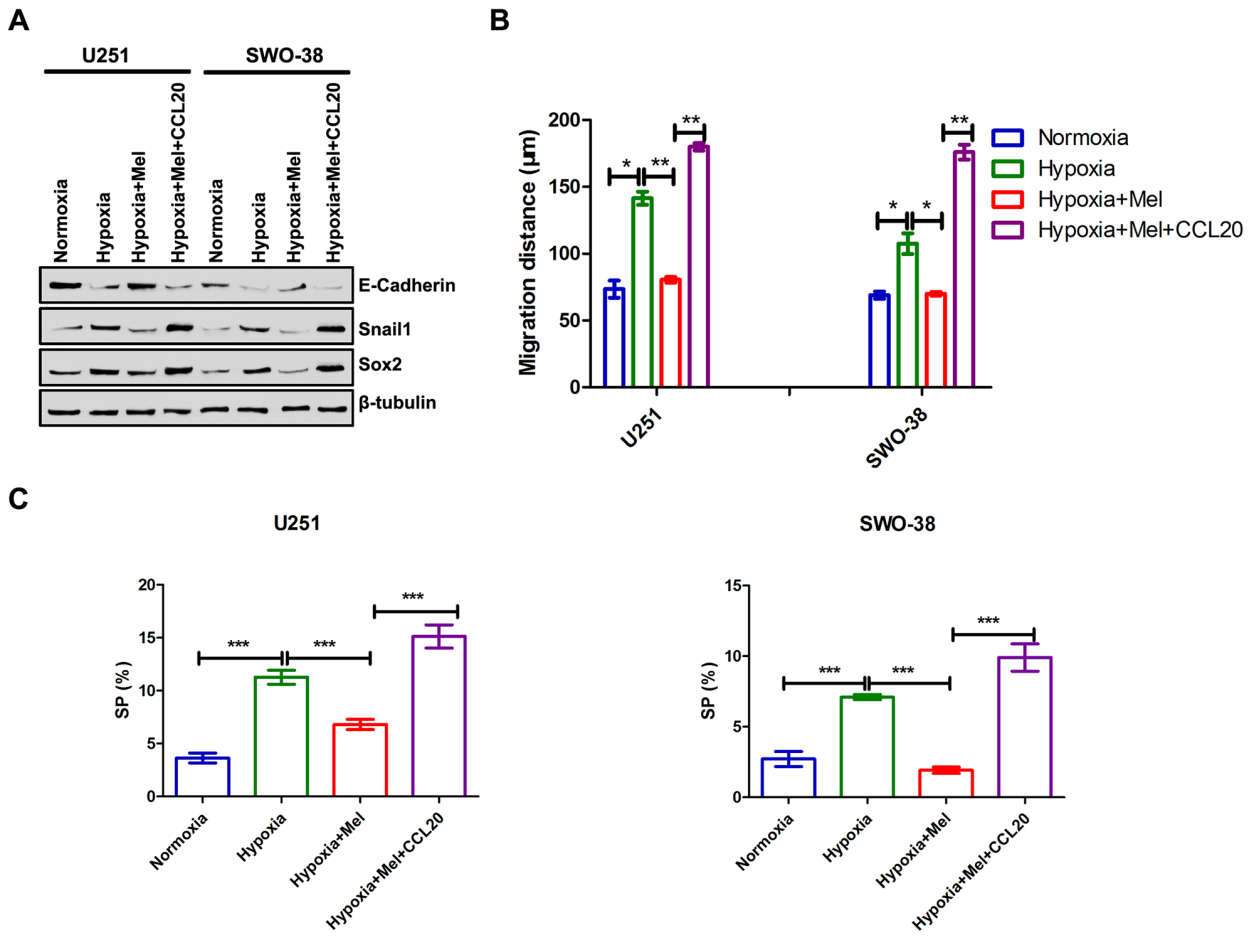
Melatonin regulated CCL20-mediated EMT, metastasis and cancer stem cell self-renewal in glioma cells U251 and SWO-38 cells treated with or without melatonin were allowed to treat with CCL20 under hypoxia. **(A)** The expression E-Caherin, Snail1 and Sox2 wasanalyzed by Western blot analysis in U251 and SWO-38 cells. β-tubulin was used as loading control. **(B)** The migration distance of U251 and SWO-38 cells was performed by wound healing assay. The shown data represent the mean±SD of triplicate determinations from three separate experiments and compared using the unpaired *t* test (^*^, P<0.05; ^**^, P<0.01). **(C)** The SP population was determined by Hoechst 33342 efflux assays in U251 and SWO-38 cells. The shown data represent the mean±SD of triplicate determinations from three separate experiments and compared using the unpaired *t* test (^***^, P<0.001).

To further evaluate the potential relationship between melatonin and CCL20 in the tumor microenvironment, co-culture assays were carried out. CCL20 could promote EMT (Figure 6A) and stemness maintenance (Figure 6A and Supplementary Figure 1) in melatonin-treated U251 and SWO-38 cells. The results from the Transwell assays also showed that the number of migrated melatonin-treated U251 and SWO-38 cells was more than only melatonin-treated cells with the co-culture of exogenous CCL20 (Figure 6B). Besides this, CCL20 also increased the abundance of SP cells in melatonin-treated cells (Figure 6C). Collectively, these results indicate that CCL20 might be an important mediator of melatonin-mediated EMT in glioma cells.

### Melatonin suppresses TGFβ/Samd-CCL20 activities and EMT through Smad7

**Figure 7:**
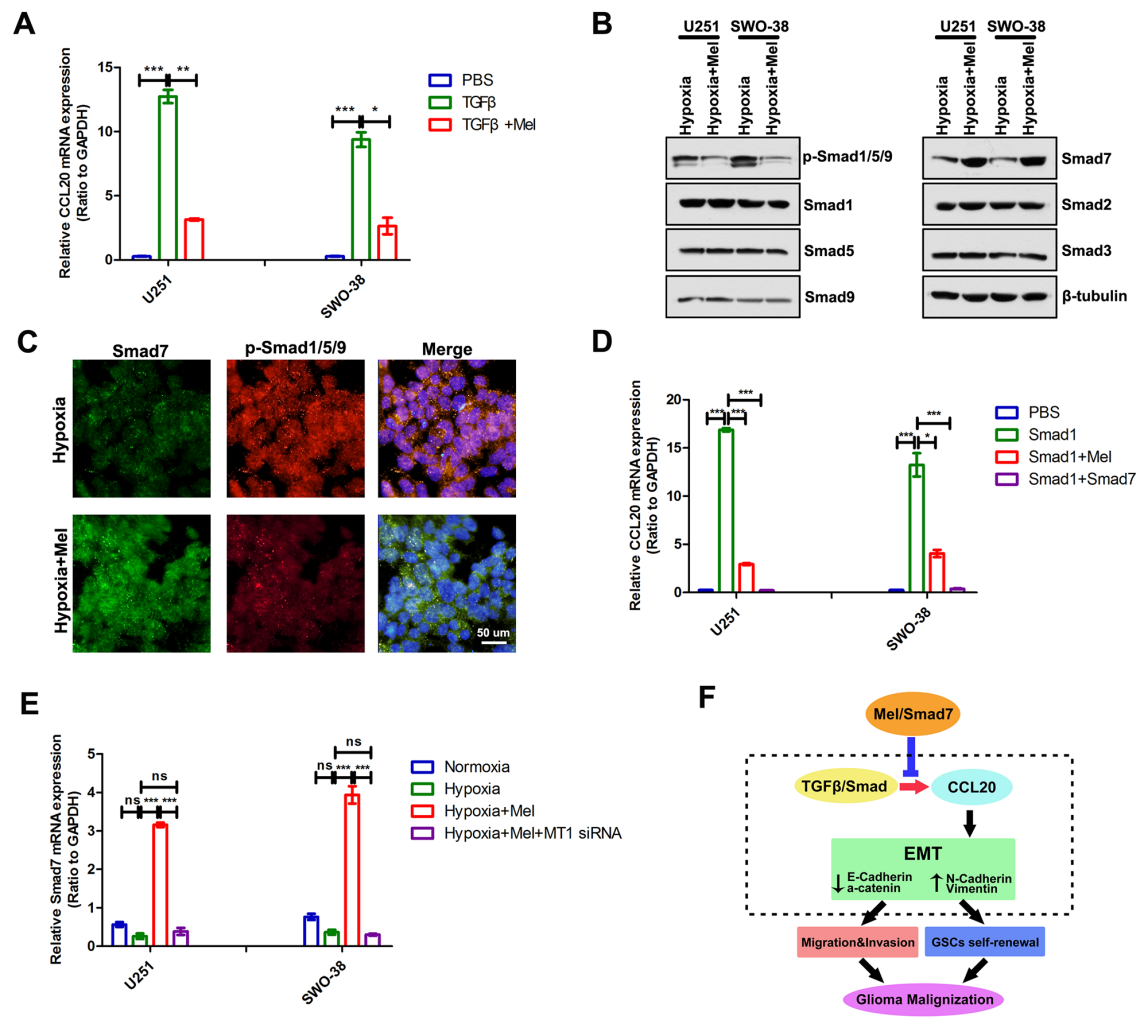
Melatonin regulated CCL20 transcriptional level through Smad7 **(A)** The expression of CCL20 was analyzed by qRT-PCR in U251 and SWO-38 treated with melatonin and TGFβ1. CCL20 mRNA level was normalized to GAPDH expression. The shown data represent the mean±SD of triplicate determinations from three separate experiments and compared using the unpaired *t* test (^*^, P<0.05; ^**^, P<0.01; ^***^, P<0.001). **(B)** The expression of R-Smads and Co-Smads of TGFβ/Smad was determined by Western blot analysis in U251 and SWO-38 cells treated with or without melatonin under hypoxia. β-tubulin was used as loading control. **(C)** The expression p-Smad1/5/9 and Samd7 was analyzed by immunofluorescence in SWO-38 cells treated with or without melatonin under hypoxia. Scale bar=50 um. **(D)** U87 and SWO-38 cells transfected with Smad1 were allowed to treat with melatonin or co-transfected with Smad7. The CCL20 mRNA level was determined by qRT-PCR. CCL20 mRNA level was normalized to GAPDH expression. The shown data represent the mean±SD of triplicate determinations from three separate experiments and compared using the unpaired *t* test (^*^, P<0.05; ^**^, P<0.01; ^***^, P<0.001). **(E)** U251 and SWO-38 cells treated with melatonin were allowed to co-transfected with melatonin receptor MT1 siRNA. The Smad7 mRNA level was determined by qRT-PCR. Smad7 mRNA level was normalized to GAPDH expression. The shown data represent the mean±SD of triplicate determinations from three separate experiments and compared using the unpaired *t* test (ns, not significant; ^**^, P<0.01; ^***^, P<0.001). **(F)** schematic diagram showing the roles of melatonin on EMT and metastasis in glioma cells. melatonin unregulated Smad7 expression to suppress TGFβ/Smad-mediated CCL20 transcript level and CCL20-induced EMT occurrence, suggesting potential anti-EMT therapeutic strategy of melatonin to overcome malignant transformation in gliomas.

Hypoxia activates the TGF-β/Smad pathway [36, 37], and a TGF-β-responsive element (i.e. Smad) is located on an upstream enhancer of the human CCL20 promoter [38, 39]. Considering that the change in CCL20 transcript levels is regulated by melatonin, we speculated that melatonin might block the signal transduction of TGFβ/Smad to suppress glioma cell EMT under hypoxia. qRT-PCR analyses showed that TGFβ significantly induced upregulation of the transcript level of CCL20, but melatonin could inhibit this upregulation in U251 and SWO-38 cells (Figure 7A). Melatonin negatively regulated the level of p-Smad1/5/9, and positively deregulated the expression of Smad7 (Figure 7B). Attesting to its specificity, melatonin exhibited little effect on the expression of Smad2 and Smad3 (Figure 7B). Immunofluorescence staining also showed that melatonin increased Smad7 expression and decreased p-Smad1/5/9 level (Figure 7C). Overexpression of Smad1 increased CCL20 mRNA level, but melatonin could inhibit this upregulation, similar to that with Smad7 overexpression (Figure 7D). Melatonin regulated Smad7 expression at transcript level; U251 and SWO-38 cells transfected with melatonin receptor MT1 siRNA could block Smad7 upregulation (Figure 7E). Thus, these results indicate that melatonin can increase expression of TGFβ inhibitory Smad, Smad7, to suppress TGFβ/Samd-CCL20 activities and EMT (Figure 7F).

## DISCUSSION

Tumor recurrence in glioma is partly attributed to increased epithelial-to-mesenchymal transition (EMT) and enhanced tumor cell dissemination in adjacent brain parenchyma [13, 14, 40]. EMT is associated with aggressive behavior, increased stem-like characteristics, and treatment resistance in malignancies [9, 11, 41]. Thus, exploring effective strategies for anti-EMT-like change in glioma invasion and recurrence will be important for glioma treatment.

EMT is a mechanism that generates cancer cells endowed with an invasive and metastatic phenotype [11, 41]. EMT also occurs during embryogenesis, and is involved in organ and tissue formation, as well as in trauma restoration and organ fibrosis and carcinogenesis. This process is mediated by the activity of growth and transcription factors, resulting in loss of the typical intercellular junction structure characteristic of epithelial cells, acquisition of mesenchymal morphology, loss of apical-basal cell polarity, and motility and invasion ability. Major molecular markers of EMT include loss of the epithelial marker E-Cadherin, and overexpression of mesenchymal markers such as N-Cadherin and Vimentin. In glioma cells, hypoxic stress induced the expression of the mesenchymal markers Vimentin, N-Cadherin, and Snail1. However, melatonin drastically inhibited hypoxia-induced expression of Vimentin, N-Cadherin, and Snail1, and reversed the E-Cadherin and α-Catenin levels. Similar to the results in *in vivo* tumor formation studies, hypoxic tumor regions showed reduced E-Cadherin levels; however, injection of melatonin substantially increased the E-Cadherin localization at the cell membrane. Studies have also demonstrated that EMT is involved in cell plasticity, a process by which non-stem cells acquire stem cell characteristics [42]. In this study, we found that hypoxia stress enriched the stem cell population, but melatonin reduced the abundance of SP cells, and antagonized stem cell characteristics. Thus, these results suggest that melatonin suppresses hypoxia-induced EMT and thus would help in developing effective strategies for glioma treatment.

Chemokines include a number of small chemoattractant proteins and belong to a superfamily of small chemotactic cytokines. These proteins are divided into four families (C, CC, CXC, and CX3C) based on the number and spacing of cysteine residues [43]. Among the CC chemokines, CCL20, also known as liver and activation-regulated chemokine (LARC), macrophage inflammatory protein-3α (MIP-3α), or Exodus-1, was initially identified in the liver [44, 45]. A large body of evidence shows that CCL20 is correlated with tumor formation, metastasis, or progression in various tumors[46]. In glioma tissues, CCL20 and its receptor, CCR6 protein levels are upregulated, compared with their expression in non-neoplastic brain tissues[47]. Additionally, the expression levels of these two proteins were significantly correlated with advanced WHO stage and low KPS scores, suggesting that CCL20 and CCR6 expression might be of clinical relevance in the aggressiveness of gliomas [47]. In our study, we used the human XL oncology array to investigate whether there was a change in the cytokine profile associated with melatonin-mediated EMT of glioma cells in hypoxia. CCL20 expression was negatively associated with E-Cadherin and positively associated with Vimentin and Snail1 under hypoxic stress. However, the levels of CCL20 decreased most dramatically in the presence of melatonin, accompanied by the blocking of EMT. Thus, these results suggest that CCL20 might be an important mediator of melatonin-mediated EMT in glioma cells.

A variety of cell signaling pathways have been implicated in the process of EMT and CCL20 expression. Hypoxia activates the TGF-β/Smad pathway, and TGF-β-responsive element (i.e. Smad) located on an upstream enhancer of the human CCL20 promoter [38, 39]. Our results show that TGFβ significantly induced the transcript level of CCL20, but melatonin can inhibit this upregulation in U251 and SWO-38 cells, suggesting melatonin might antagonize TGFβ/Smad to inhibit CCL20 expression. In an earlier study, we showed that melatonin can inhibit R-Smad phosphorylation and its transcript activity [48]. Further studies showed that melatonin negatively regulates the level of p-Smad1/5/9, and positively deregulates the expression of Smad7. Mechanistically, SMAD7 serves as scaffold to recruit the SMAD-specific E3 ubiquitin protein ligase 2 (SMURF2) to the TGF β receptor complex to facilitate receptor polyubiquitination and complex degradation [49, 50]. Moreover, the binding of SMAD7 at TβRI occurs at the same site as the R-Smads, further limiting R-Smad activation [49, 50]. Thus, melatonin induces the TGFβ inhibitory Smad, Smad7, to suppress TGFβ/Smad-CCL20 activities and EMT. How melatonin regulates Smad7 level? Our results show that melatonin regulates Smad7 expression at the transcript level, because transfection of U251 and SWO-38 cells with siRNA targeting the melatonin receptor MT1 blocks Smad7 upregulation. Cross-talk between Smad7 and ERK signaling transduction, and melatonin can activate ERK pathway. Therefore, we speculate that melatonin transcriptionally regulates Smad7 expression through ERK activation. This specific transcriptional regulation of Smad7 by melatonin needs to be further studied.

In summary, our study provides data that clearly show that melatonin induces Smad7 expression leading to suppression of TGFβ/Smad-CCL20 activities and EMT in gliomas, indicating that melatonin may be used as a novel therapeutic to treat malignant transformation in gliomas.

## MATERIALS AND METHODS

### Chemicals and antibodies

Lipofectamine 3000 transfection reagent, TRIzol LS, and TGFβ1 were purchased from Invitrogen. Phospho-Smad1/5/9, Smad1, Smad2, Smad3, Smad4, Smad7, and Smad9 antibodies were from Cell Signaling Technology. E-Cadherin, α-Catenin, N-cadherin, Vimentin, Snail1, and HIF1α antibodies were from Abcam. Sox2, Oct4, CD133, and β-tubulin antibodies were from Millipore. Melatonin was purchased from Sigma. CCL20 was obtained from Peprotec. Smad1 and Smad7 plasmid constructs were purchased from Addgene.

### Cell culture

U251 and SWO38 glioma cell lines were purchased from the Cell Bank of Type Culture Collection of the Chinese Academy of Sciences, Shanghai Institute of Cell Biology, Chinese Academy of Sciences (Shanghai, China), where they were characterized by DNA fingerprinting and isozyme detection. Cell lines were revived every 3 to 4 months, and cultured in Dulbecco’s Modified Eagle’s Medium (Hyclone) supplemented with 10 % fetal bovine serum ( FBS) and 1 % (100X) penicillin/streptomycin (Hyclone). For hypoxia treatment, the cells were exposed to 1.0 % O2 in a hypoxic chamber (Thermal Tech) for the indicated time period.

### *In vivo* tumor formation

Nude mice were subcutaneously injected at the upper left flank region with 0.1 ml of cell suspension containing 5 × 10^6^ cells from either the U251 or SWO-38 cell line. Nude mice were injected with 10 mg/kg melatonin every five days through the tail vein. Tumor growth was evaluated by measuring the length and width of the tumor mass at the inoculation site. After six weeks, the tumor-bearing mice were sacrificed and their lungs were excised. Animal experiments were performed according to the guidelines of the Animal Use and Care Committees at Hefei Institutes of Physical Science, CAS.

### Wound healing assay, cell invasion assay, and cell motility assay

Scratch wound healing assay was performed to assess cell migration. In brief, 3 × 10^4^ U251 or SWO-38 cells were cultured in a 24-well plate for 24 h. After a tight cell monolayer was formed, the cells were incubated with serum-free medium for 24 h and the cell monolayer was wounded with a plastic pipette tip. The remaining cells were washed twice with fresh medium to remove cell debris, and further cultured. At the indicated time points, the migrant cells at the wound front were photographed with a microscope.

The cell invasive assay was performed as described earlier[21]. Briefly, a cell suspension of 1 × 10^5^ U251 or SWO-38 cells in 100 μl of serum-free DMEM was placed into the upper compartment of a Boyden chamber (Corning) precoated with Matrigel, and 600 μl defined medium was added to the lower compartment as a chemoattractant. After incubating for 48 h, the cells that failed to penetrate the filters were gently removed by cotton swabs. The invading cells in the membrane were fixed with 4% formaldehyde in PBS, stained in crystal violet for 10 min, and then counted under a light microscope. Cell motility assay was performed similarly except that an uncoated filter was used and the incubation time was 18 h.

### Hoechst 33342 staining, and flow cytometry analysis side population (SP) cells

Cells were washed with PBS, detached from the culture dish with trypsin and EDTA, pelleted by centrifugation, and resuspended in DMEM containing 2 % FBS at a concentration of 1×10^6^ cells/ml. The cells were incubated with 5 μg/ml Hoechst 33342 (Sigma, St, Louis, MO) for 60 min at 37°C. After staining, the cells were centrifuged and resuspended in HBSS (Invitrogen) containing 1 μg/ml propidium iodide and maintained at 4°C for flow cytometry analysis and sorting. Cell analysis was performed on a Beckman cytometer (CytoFLEX FCM; Beckman).

### Sphere-formation assay

Single-cell suspensions were plated in ultralow attachment 96-well plates (Corning) at different densities of viable cells. Cell were grown in a serum-free epithelial growth medium (EGM), supplemented with 1:50 B27 (Invitrogen), 10 ng/ml EGF, 10 ng/ml basic fibroblast growth factor (bFGF) (BD), and 10 μg/ml heparin (Sigma). The numbers of spheroids were counted after 7 days.

### Human cytokine antibody array

The medium from SWO-38 cells was incubated with the human XL oncology array membrane (ARY026; R&D Systems) at 4°C overnight. After washing to remove unbound material, streptavidin-HRP and chemiluminescent detection reagents (Thermo) were sequentially added. Light was produced at each spot in proportion to the amount of bound analyte. Data was captured by exposure to X-ray films. Array signals from the scanned X-ray film images were analyzed using ImageJ. The results were expressed as fold changes above or below the unexposed cultures.

### Western blot analysis

Cells were lysed in lysis buffer and total protein content was determined by the Bradford method. Thirty micrograms of protein was separated by SDS-PAGE under reducing conditions and blotted onto PVDF membranes (Millipore). The membranes were probed with specific antibodies, washed, and then probed with respective secondary peroxidase-conjugated antibodies. The bands were visualized by chemoluminescence (Thermo).

### qRT-PCR assay

Total RNA was extracted using TRIzol reagent (Invitrogen), from which cDNA was synthesized using SuperScript II Reverse Transcriptase (Invitrogen). qRT-PCR and data collection were performed with an Roche LightCycler 480 detection system. The primers used for the amplification of the indicated genes are listed below: E-Cadherin, 5’-cggacgatgatgtgaacacc-3’ and 5’-ttgctgttgtgcttaacccc-3’; α-Catenin, 5’-attagtggggctgccttgat-3’ and 5’-gtccctggtcttcttggtca-3’; N-Cadherin, 5’-cggtttcatttgagggcaca-3’ and 5’-ttggagcctgagacacgatt-3’; Snail1, 5’-ccccaatcggaagcctaact-3’ and 5’-gacagagtcccagatgagca-3’; Vimentin, 5’-tgcaggctcagattcaggaa-3’ and 5’-ctccggtactcagtggactc-3’; CCL20, 5’-ctcctggctgctttgatgtc-3’ and 5’-atttgcgcacacagacaact-3’; Smad7, 5’-tagccgactctgcgaactag-3’ and 5’-cactctcgtcttctcctccc-3’; and GAPDH, 5’-ccagaacatcatccctgcct-3’ and 5’-0cctgcttcaccaccttcttg-3’.

### Statistical analysis

Data were recorded as mean ± SD. Comparisons between different groups were undertaken using the Student’s two-tailed *t* test. The limit of statistical significance was *P* < 0.05. Statistical analysis was performed using the SPSS/Win11.0 software.

## SUPPLEMENTARY MATERIALS FIGURE


