# Supplementary material for: Melatonin attenuates hypoxia-induced epithelial-mesenchymal transition and cell aggressive via Smad7/ CCL20 in glioma
Source: Oncotarget. 2017 Aug 24;8(55):93580–92. doi: 10.18632/oncotarget.20525 (PMC5706820; doi:10.18632/oncotarget.20525)
Supplement: Supplementary file 1 [file oncotarget-08-93580-s001.pdf]

Melatonin attenuates hypoxia-induced epithelial-mesenchymal transition and cell aggressive via Smad7/ CCL20 in glioma

SUPPLEMENTARY MATERIALS

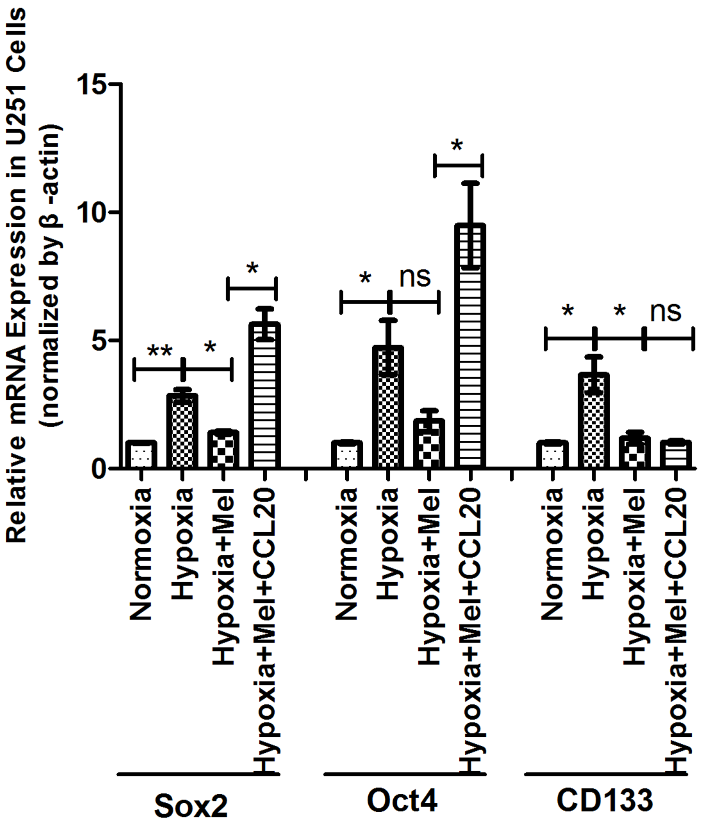

Supplementary Figure 1: U251 and SWO-38 cells treated with or without melatonin were allowed to treat with CCL20 under hypoxia. The expression Sox2, Oct4 and CD133 was analyzed by by qRT-PCR in U251. Sox2, Oct4 and CD133 mRNA level was normalized to β-actin expression. The shown data represent the mean±SD of triplicate determinations from three separate experiments and compared using the unpaired t test (ns, not significant; *, P<0.05; **, P<0.01; ***, P<0.001).
